# Optimization of Statin-Loaded Delivery Nanoparticles for Treating Chronic Liver Diseases by Targeting Liver Sinusoidal Endothelial Cells

**DOI:** 10.3390/pharmaceutics15102463

**Published:** 2023-10-14

**Authors:** Mar Gil, Lareen Khouri, Imma Raurell, Diana Rafael, Fernanda Andrade, Ibane Abasolo, Simo Schwartz, María Martínez-Gómez, María Teresa Salcedo, Juan Manuel Pericàs, Diana Hide, Mingxing Wei, Norman Metanis, Joan Genescà, María Martell

**Affiliations:** 1 Liver Disease Group, Liver Unit, Vall d’Hebron Research Institute (VHIR), Vall d’Hebron University Hospital, Vall d’Hebron Hospital Campus, Universitat Autonòma de Barcelona (UAB), 08035 Barcelona, Spain; mar.gilsoler@gmail.com (M.G.); joan.genesca@vallhebron.cat (J.G.); 2 Institut of Chemistry, Casali Center for Applied Chemistry, The Center for Nanoscience and Nanotechnology, The Hebrew University of Jerusalem, Jerusalem 91904, Israel; 3 Centro de Investigación Biomédica en Red de Enfermedades Hepáticas y Digestivas (CIBERehd), Instituto De Salud Carlos III, 08035 Barcelona, Spain; 4 Clinical Biochemistry, Drug Delivery and Therapy Group (CB-DDT), Vall d’Hebron Research Institute (VHIR), Vall d’Hebron University Hospital, Vall d’Hebron Hospital Campus, 08035 Barcelona, Spain; 5 Centro de Investigación Biomédica en Red de Bioingenería, Biomateriales y Nanomedicina (CIBER-BBN), Instituto De Salud Carlos III, 08035 Barcelona, Spain; 6 Departament de Farmàcia i Tecnologia Farmacèutica i Fisicoquímica, Facultat de Farmàcia i Ciències de l’Alimentació, Universitat de Barcelona (UB), 08007 Barcelona, Spain; 7 Clinical Biochemistry Service, Vall d’Hebron University Hospital, Vall d’Hebron Barcelona Hospital Campus, 08035e Barcelona, Spain; 8 Pathology Department, Vall d’Hebron University Hospital, Vall d’Hebron Barcelona Hospital Campus, 08035 Barcelona, Spain; 9 Cellvax, SAS Villejuif Bio Park, 93230 Villejuif, France; ming@cellvax-pharma.com

**Keywords:** chronic liver disease, liver fibrosis, peptide ligands, portal hypertension, therapy, targeted polymeric micelles

## Abstract

In this study, we developed functionalized polymeric micelles (FPMs) loaded with simvastatin (FPM-Sim) as a drug delivery system to target liver sinusoidal endothelial cells (LSECs) for preserving liver function in chronic liver disease (CLD). Polymeric micelles (PMs) were functionalized by coupling peptide ligands of LSEC membrane receptors CD32b, CD36 and ITGB3. Functionalization was confirmed via spectroscopy and electron microscopy. In vitro and in vivo FPM-Sim internalization was assessed by means of flow cytometry in LSECs, hepatocytes, Kupffer and hepatic stellate cells from healthy rats. Maximum tolerated dose assays were performed in healthy mice and efficacy studies of FPM-Sim were carried out in bile duct ligation (BDL) and thioacetamide (TAA) induction rat models of cirrhosis. Functionalization with the three peptide ligands resulted in stable formulations with a greater degree of in vivo internalization in LSECs than non-functionalized PMs. Administration of FPM-Sim in BDL rats reduced toxicity relative to free simvastatin, albeit with a moderate portal-pressure-lowering effect. In a less severe model of TAA-induced cirrhosis, treatment with FPM-CD32b-Sim nanoparticles for two weeks significantly decreased portal pressure, which was associated with a reduction in liver fibrosis, lower collagen expression as well as the stimulation of nitric oxide synthesis. In conclusion, CD32b-FPM stands out as a good nanotransporter for drug delivery, targeting LSECs, key inducers of liver injury.

## 1. Introduction

Chronic liver disease (CLD) is responsible for more than 2 million annual deaths worldwide [1]. Progressive CLD is caused by the continuous production and deposition of extracellular matrix components resulting in significant hepatic fibrosis and nodular distribution of the liver parenchyma, leading to organic dysfunction and liver failure [2,3]. Portal hypertension is an important complication of advanced CLD and is the consequence of a marked increase in intrahepatic vascular resistance (IHVR) together with an increased hepatic vascular tone [4]. This increased vascular tone is strongly related to alterations in liver sinusoidal endothelial cells (LSECs) that, upon chronic injury, exhibit an imbalance of vasoactive molecules favoring vasoconstriction and increasing IHVR.

LSECs play a major role as sensors and drivers of CLD [5]. Under physiological conditions, LSECs are responsible for maintaining hepatic homeostasis, metabolite transport and vascular tone, enhancing hepatocytes exposure to macromolecules from the portal circulation. Furthermore, LSECs possess anti-inflammatory and anti-fibrogenic properties by preventing the activation of Kupffer cells and hepatic stellate cells (HSCs) [6]. However, under pathological conditions, LSECs dedifferentiate, becoming a common vascular endothelial phenotype and losing specialized functions [7,8]. Therefore, it is crucial to maintain the LSEC specialized phenotype to preserve hepatic function; this might be achieved by means of the direct delivery of vasoprotective drugs.

Statins, originally designed as inhibitors of the enzyme 3-hydroxy-3-methylglutaryl-co-enzyme A reductase (HMG-CoA reductase) of the cholesterol biosynthesis pathway [9,10], have been widely used for lipid-lowering purposes with a good safety profile in cardiovascular diseases. However, although the beneficial effects of statins in the cirrhotic liver have been demonstrated, their use in CLD has not yet been consolidated. Furthermore, different animal models of cirrhosis have shown that the use of statins (e.g., simvastatin, atorvastatin) exerts vasoprotective and antifibrotic effects, improving LSEC dysfunction, reducing HSC activation and achieving hepatic fibrosis regression. These remarkable beneficial effects were mainly dependent on the up-regulation of Krüppel-like factor 2 (KLF2) in hepatic cells after statins treatment [11,12] by enhancing the expression of their target genes, such as eNOS (endothelial nitric oxide synthase), or inhibiting the vasoconstrictive RhoA/ROCK (Ras homolog family member A/Rho-associated protein kinase) axis, among other pathways [13,14]. However, statins are not free of adverse effects, mainly including muscular but also hepatic toxicity [15,16]. As an example, in a previous study in a rat model with bile elimination impairment (bile duct ligation model, BDL) mimicking severely impaired liver function, the adverse events of simvastatin were magnified, reaching significant levels of mortality when using high doses [13]. Therefore, the dose-dependent side effects of statins limit their efficacy, especially in populations with a higher risk of toxicity, such as patients with advanced CLD [17].

To overcome this limitation, we produced Pluronic-based polymeric micelles (PMs), loaded with chemically activated simvastatin [18]. Briefly, the nanoparticle biodistribution was mainly hepatic and no signs of toxicity were detected. After one week of administration in the BDL model, these nanoparticles demonstrated superior effects compared to free simvastatin in reducing portal pressure, with a significant nanoparticle accumulation in LSECs, although they were also found in Kupffer cells, suggesting a possible loss of efficacy due to their clearance by macrophages. Therefore, it was proposed that an improvement to the formulation by means of functionalization with specific ligands was needed to increase the effect of nanoparticles in LSECs in a more specific manner [19].

One of the outstanding properties of Pluronic PMs is their ability to be functionalized by adding peptide or protein molecules for active targeting to their hydrophilic surface to enhance drug delivery to specific sites in the body or specific receptors on cells. By doing this, the therapeutic efficacy of the cargo can be maximized while reducing systemic toxicity compared with untargeted micelles [20]. When the ligands conjugated to PMs bind to their specific receptors on the cell membrane, endocytic internalization of those nanoparticles is promoted. Interestingly, LSECs have one of the highest endocytic capacities in the human body, providing an advantage for specific targeting [21].

This study explores the development of nanoparticles specifically targeting LSECs for the delivery of simvastatin. We tested different peptides recognizing the binding sites of (i) the specific LSEC Fcγ-receptor IIb, expressed mainly in physiological conditions, (ii) the transmembrane glycoprotein thrombospondin receptor, unaltered during LSEC capillarization and (iii) the integrin α_V_β_3_ receptor, highly expressed in dysfunctional LSECs.

## 2. Material and Methods

### 2.1. Reagents

Fluorenylmethyloxycarbonyl protecting group (Fmoc)-*L*-amino acids were obtained from CSBio (Menlo Park, CA, USA) or Matrix Innovation Inc. (Quebec, QC, Canada). *O*-(1*H*-6-chlorobenzotriazole-1-yl)-1,1,3,3-tetramethyluronium hexafluorophosphate (HCTU) was obtained from Luxembourg Bio Technologies Ltd. (Ness Ziona, Israel). Solvents for peptide synthesis such as piperidine, *N,N*-dimethylformamide (DMF), N,N-diisopropylethylamine (DIPEA), dichloromethane (DCM), trifluoroacetic acid (TFA), trisopropylsilane and acetonitrile were purchased from Bio-Lab Ltd. (Jerusalem, Israel). Ultrapure guanidinium chloride (GdmCl; Apollo Scientific Ltd., Stockport, UK) used in the buffer for dissolving the peptides. Pluronic F127 was provided by BASF (Ludwigshafen, Germany). Pure simvastatin, fluorescein 5-isothiocyanate (FITC), 5-([4,6-dichlorotriazin-2-yl])aminofluorescein (5-DTAF), maleic anhydride, 1-ethyl-3-(3-dimethylaminopropyl)carbodiimide (EDC), 4′,6-diamidino-2-phenylindole (DAPI) and propidium iodide solution were obtained from Sigma-Aldrich (Merck KGaA, Darmstadt, Germany). Other reagents such as the culture media, phosphate-buffered saline (PBS), trypsin, fetal bovine serum (FBS) and bovine serum albumin (BSA), were provided by Biowest (Nuaillé, France). All TaqMan probes were obtained from Applied Biosystems (Thermo Fisher Scientific, Waltham, MA, USA).

### 2.2. Animals

All animal procedures were conducted in accordance with European Union legislation on the protection of animals used for scientific purposes (Directive 2010/63/EU revising Directive 86/609/EEC), approved by the Animal Research Ethics Committee of the Vall d’Hebron Institut de Recerca (Barcelona, Spain).

Male Sprague Dawley CD rats (Charles River Laboratories, Saint-Germain-sur-l’Arbresle, France) were used to generate two models of CLD with portal hypertension and cirrhosis; intrahepatic portal hypertension was induced via secondary biliary cirrhosis, performing a common BDL for a period of 4 weeks on rats weighing 200–220 g, or via intraperitoneal injection of 250 mg/kg of thioacetamide (TAA) 2 days/week over a period of 8 weeks to rats weighing 125–150 g at baseline and weighed weekly to adjust the dose to the weight gain. Only male rats were used because female estrogen hormones modify hemodynamic parameters, increasing or decreasing their values randomly and unrelated to liver disease [22]. All animals were housed under a constant temperature of 22 ± 2 °C and 50% humidity in a controlled 12/12 h light/dark cycle. They were fed ad libitum with a grain-based chow (SAFE 150; SAFE Complete Care Competence, Rosenberg, Germany) and had free access to water.

In addition, male BALB/cAnNRj mice (Janvier Labs, Le Genest-Saint-Isle, France) aged 5–6 weeks were used for the maximum tolerated dose assay performed at Cellvax facilities (Villejuif, France). Animal health status was specific and opportunistic pathogen-free (SOPF), and they were housed in polyethylene cages (<5 mice/cage) in a climate- and light-controlled environment in accordance with Cellvax’s approved standard operating procedures.

### 2.3. Simvastatin Activation

Prior to encapsulation or in vitro treatment, commercial pure simvastatin needs to be activated from its inactive lactone form to its β-hydroxyacid active form [23]. For this purpose, 8 mg of simvastatin (0.019 mM) was dissolved in 0.2 mL of pure ethanol, with the subsequent addition of 0.3 mL of 0.1 N sodium hydroxide (NaOH). The solution was heated at 50 °C for 2 h and neutralized with hydrochloric acid (HCl) to pH 7.0. The resulting solution was brought to a final volume of 1 mL with distilled water, and aliquots were stored at −80 °C until use or lyophilized.

### 2.4. Synthesis of Peptide Ligands

All peptides were prepared via the solid-phase peptide synthesis (SPPS) technique [24] using standard Fmoc-*L*-amino acids on a CS136X peptide synthesizer (CSBio, Mountain View, CA, USA). Fmoc deprotection was performed twice using 20% piperidine in DMF for 5 min. The carboxylic acid functional group (-COOH) of each amino acid was activated with the coupling reagent HCTU in the presence of DIPEA for 5 min and added to the resin for coupling with constant shaking for 30 min at room temperature (RT). The resin was washed -three times with DMF and DCM, dried and cleaved with a cocktail of TFA/trisopropylsilane/water (ratio 94:3:3). After cleavage, the resin was removed by means of filtration, washed twice with neat TFA and bubbled with nitrogen for TFA removal. Afterwards, cold diethyl ether was added to precipitate the peptide, centrifuged (4000 rpm, 10 min) and ether was decanted. Cleaved peptide was dissolved in 0.1% TFA in GdmCl and lyophilized.

For the synthesis of fluorescently labelled peptides, Fmoc-L-Lys(Alloc)-OH was coupled on the C-terminus of the peptide chain to label it with FITC, allowing the free amine (NH_2_) of Lys to react with the fluorochrome.

### 2.5. Purification and Characterization of Peptides

The analytical analyses RP-HPLC were performed on a reverse-phase Waters Alliance HPLC with UV detector (220 nm and 280 nm) using an X-Bridge C18 column (3.5 µm, 130 Å, 4.6 × 150 mm). Preparative RP-HPLC was performed on a Waters LCQ150 system (XSelect PPeptide CSH^TM^ C18 column, 3.5 µm, 130 Å, 30 × 250 mm). Linear gradients of acetonitrile with 0.1% TFA (buffer B) and water with 0.1% TFA (buffer A) were used for all systems to elute peptides. The flow rates were 1 mL/min (analytical) and 10 mL/min (C4 preparative).

Lyophilized crude peptides were dissolved in acetonitrile in water with 0.1% TFA and purified by means of prep RP-HPLC, and peaks were collected and characterized by means of electrospray ionization mass spectrometry (ESI-MS) performed on a LCQ Fleet Ion Trap Mass Spectrometer (Thermo Scientific, Thermo Fisher Scientific, USA). Peptide masses were calculated from the experimental mass-to-charge ratios (*m*/*z*) from all the observed multiply charged species of peptides.

### 2.6. Synthesis of Carboxylated Polymer for Functionalization

For the synthesis of functionalized polymeric micelles (FPMs), Pluronic F127 was carboxylated (F127-COOH) by means of the maleic anhydride method [25] to assist with the peptide ligand conjugation. F127 and maleic anhydride (ratio 1:11) were dissolved in distilled chloroform and allowed to react for 24 h under stirring at 70 °C in a condensation system to avoid any loss of solvent. The solution was concentrated and poured twice into an excess amount of iced cold diethyl ether to precipitate the reaction product. F127-COOH was dried via vacuum dehydration and collected as a white powder.

### 2.7. Production of Polymeric Micelles (PMs)

The synthesis of PMs was based on the thin-film hydration technique. Briefly, Pluronic F127 polymer was weighed and dissolved in an organic mixture of methanol/ethanol (1:1), which was removed under vacuum in a rotary evaporator and the formed thin film was left to dry overnight (O/N) at RT to eliminate any remaining solvent. The film was hydrated with PBS at RT and the aqueous solution was vortexed for 5 min, allowing polymers to self-assemble to form micelles. For PM functionalization, micelles were produced with a mixture of F127 and F127-COOH polymers (ratio 8:2), incubated with EDC dissolved in water (polymer/EDC ratio 1:1.5) and stirred for 30 min at RT to activate COOH groups. To conjugate the activated PMs with the peptide ligands, the native chemical ligation technique was utilized, where synthesized peptides, modified with a Cys residue at the N-terminus, were solubilized in PBS, added to the PM solution (peptide/PM ratio 1:100) and incubated under stirring for 2 h at RT. The obtained dispersion of PMs or FPMs was filtered through a 0.22 µm syringe filter for sterilization and the removal of eventual aggregates. All types of nanoparticles were lyophilized for long-term storage at RT until use.

For internalization studies, fluorescent micelles were synthesized using 10% 5-DTAF-labelled polymer obtained via the conjugation of Pluronic F127 polymer with 5-DTAF in an aqueous medium via nucleophilic aromatic substitution through an addition–elimination mechanism.

For the synthesis of drug-loaded micelles, activated simvastatin was dissolved at the desired concentration (20 or 40 mg/mL) in the organic methanol/ethanol mixture together with the polymer before the solvent evaporation step.

### 2.8. Physicochemical Characterization of PM

To confirm its correct functionalization, FPMs were analyzed by means of Fourier-transform infrared spectroscopy (FTIR) in the Preparation and Characterization of Soft-Materials Services at Institut de Ciència de Materials de Barcelona (Barcelona, Spain) using a Spectrum One FT-IR Spectrometer (PerkinElmer, Inc., Waltham, MA, USA), with an energy range of 450–4000 cm^−1^, equipped with the Universal Attenuated Total Reflectance (UATR) accessory.

Particles’ mean hydrodynamic diameter and polydispersity index were measured by means of dynamic light scattering (DLS), and zeta potential was measured via laser Doppler micro-electrophoresis using a Zetasizer Nano (Malvern Instruments, Malvern, UK) with an angle of 173° in a measurement range of 0.3 nm–10 μm and sensitivity of 0.1 mg/mL.

Particle shape and size were observed by means of transmission electron microscopy using the high-performance and high-contrast 120 kV JEM-1400Flash Electron Microscope (JEOL Ltd., Tokyo, Japan) from the Electron Microscopy Service at Univeristat Autònoma de Barcelona (Cerdanyola del Vallès, Spain). For their visualization, samples were placed on a copper carbon-coated grid and negatively stained with uranyl acetate for 1 min at RT. Gatan software V 2.32.888.0 (Gatan, Inc., Pleasanton, CA, USA) was used to process information and obtain measures from transmission electron microscopy images.

### 2.9. Encapsulation Efficiency

The encapsulation efficiency (EE) of simvastatin for each formulation was calculated according to Equation (1). The free drug present in the aqueous phase of the micelle synthesis was obtained via centrifugation with filtration (10,000 rpm, 10 min, 4 °C) using a Nanosep Centrifugal Device with Omega Membrane 10K (Pall Corporation, Port Washington, NY, USA) and analyzing the filtrate via UPLC-MS/MS (Xevo TQ Absolute; Waters Corporation, Los Angeles, CA, USA). All experiments were performed in triplicate at 20 °C.

(*) EE (%) = [(Total amount of simvastatin − Free simvastatin in filtrate)/(Total amount of simvastatin)] × 100(1)

### 2.10. In Vitro Drug Release Assay

The in vitro release profile of simvastatin from FPMs was assessed via the regular dialysis method, placing FPM-Sim (20 mg/mL) inside a Spectra/Por Float-A-Lyzer G2 dialysis device (MWCO 20 kDa; Spectrum Laboratories, Inc., Rancho Dominguez, CA, USA) immersed in a PBS pH 7.4 solution (1:100 dilution) and maintained at 37 °C under stirring. A 500 µL sample of released media was collected at predetermined time points (0.25, 0.5, 1, 3, 6, 9, 12, 24, 48 and 72 h) for simvastatin quantification via UPLC-MS/MS (Xevo TQ Absolute). This volume was replaced with fresh buffer at each time point. All formulations were analyzed in duplicate.

### 2.11. Isolation and Culture of Primary Rat Liver Cells

All liver cells were isolated from healthy rats as follows.

#### 2.11.1. LSECs and Kupffer cells

The liver was perfused through the portal vein for 10 min at a flow rate of 20 mL/min at 37 °C with Hanks’ Balanced Salt Solution (HBSS; Sigma-Aldrich, Merck KGaA, Darmstadt, Germany) without calcium and magnesium containing 12 mM HEPES (pH 7.4), 0.6 mM EGTA, 0.23 mM BSA and 1% heparin. Next, the liver was perfused with 0.15 mg/mL collagenase A (Roche, Merck KGaA, Darmstadt, Germany) in HBSS containing 12 mM HEPES (pH 7.4) and 4 mM calcium chloride dihydrate (CaCl_2_·2H_2_O) for 30 min at a flow rate of 5 mL/min at 37 °C, then excised and ex vivo digested with the same buffer for 10 min at 37 °C in constant agitation. Cells were passed through a 100 μm nylon filter, collected in cold Krebs’ buffer and centrifuged at 50× *g* for 3 min at 4 °C to eliminate hepatocytes. The supernatant was then centrifuged at 800× *g* for 10 min, and the pellet was resuspended in cold PBS to be centrifuged at 800× *g* for 25 min through a two-step 25–50% Percoll gradient (Sigma-Aldrich, Merck KGaA, Darmstadt, Germany) at 4 °C. The interface of the gradient enriched in LSECs and Kupffer cells was collected, rinsed with PBS and centrifuged at 800× *g* for 10 min. The pellet was resuspended in tempered correctly supplemented RPMI medium (10% FBS, 1% *L*-glutamine, 1% penicillin-streptomycin, 1% amphotericin B, 0.1 mg/mL heparin and 0.05 mg/mL endothelial cell growth supplement), seeded into a culture dish and incubated for 30 min (37 °C, 5% CO_2_). Kupffer cells were separated from LSECs via their selective adherence to the non-coated dish, being washed with PBS and maintained in RPMI at 37 °C (5% CO_2_). Non-adherent LSECs were collected, seeded into a collagen-coated culture dish and incubated for 45 min (37 °C, 5% CO_2_). Finally, they were washed with PBS and maintained in RPMI (37 °C, 5% CO_2_) [26].

#### 2.11.2. Hepatocytes

After centrifuging the digested liver filtrate at 50× *g* for 3 min at 4 °C, hepatocytes were found in the pellet, which were resuspended in cold PBS. This step was repeated 2–3 times to rinse the cells from cellular debris. Cells were then resuspended in tempered supplemented Dulbecco’s modified Eagle’s medium (DMEM) (10% FBS, 1% *L*-glutamine, 1% penicillin-streptomycin, 1% amphotericin B, 1 μM dexamethasone and 0.002% insulin) and filtered with a 100 μm filter to eliminate residues of liver tissue. Hepatocytes were planted in a collagen-coated culture dish and incubated at 37 °C and 5% CO_2_. After 4 h, cells were washed with PBS and cultured in maintenance DMEM medium (2% FBS, 1% *L*-glutamine, 1% penicillin-streptomycin, 1% amphotericin B, 1 nM dexamethasone and 0.002% insulin) [26].

#### 2.11.3. Hepatic Stellate Cells

HSCs were isolated by perfusing the rat liver through the portal vein with Gey’s Balanced Salt Solution (GBSS; Sigma-Aldrich, Merck KGaA, Darmstadt, Germany) at a flow rate of 20 mL/min at 37 °C. The liver was then perfused with 1.5 mg/mL pronase E (Roche, Merck KGaA, Germany), 0.15 mg/mL collagenase A and 0.05 mg/mL DNase I (Roche, Merck KGaA, Germany) in GBSS solution for 30 min at a flow rate of 5 mL/min at 37 °C. The digested liver was excised and digested ex vivo, also with pronase E (0.4 mg/mL) and collagenase A and DNase I (0.1 mg/mL) for 10 min at 37 °C in agitation. The resulting suspension was filtered through a 100 μm nylon filter and centrifuged at 50× *g* for 4 min at 21 °C. The supernatant was centrifuged at 800× *g* for 5 min to eliminate the hepatocytes, and the obtained pellet was resuspended in GBSS and centrifuged in Optiprep Density Gradient Medium (11.5%) (Sigma-Aldrich, Merck KGaA, Darmstadt, Germany) at 1400× *g* for 21 min. The fraction enriched in HSCs was collected, rinsed with GBBS and centrifuged at 800× *g* for 5 min. The pellet was resuspended in tempered correctly supplemented Iscove’s modified Dulbecco’s medium (IMDM) (10% FBS, 1% *L*-glutamine, 1% penicillin-streptomycin and 1% amphotericin B), seeded into a culture dish and incubated O/N at 37 °C (5% CO_2_). The next day, HSCs were washed with PBS and maintained in IMDM (37 °C, 5% CO_2_) [27].

### 2.12. Cellular Uptake of Peptide Ligands and Micelles

To determine the in vitro uptake of peptide ligands and FPM, healthy LSECs were seeded into a 96-well plate and treated with 15 μl of FITC-peptide ligands (0.75 mg/mL) or 10 μL of 5-DTAF-labelled FPMs (100 mg/mL polymer) at different time points: from 1 min to 1 h (peptide ligands) or 4 h (FPMs). As negative control, some LSECs were not treated. Next, cells were washed with PBS and for 5–10 min at 37 °C. Once the cells were detached after incubation with 30 μL of trypsin 10×, 120 μL of PBS at 5% FBS with DAPI (1:1000 dilution) was added per well and the plate was read by means of flow cytometry on a BD LSRFortessa Cell Analyzer (BD, Franklin Lakes, NJ, USA), detecting the percentage of positive cells for the fluorescent staining of peptide ligands or FPMs. Each condition was analyzed in triplicate.

For the in vivo uptake of micelles, healthy rats received an intravenous dose of 5-DTAF-labelled PMs or FPMs (100 mg/kg of polymer). Untreated rats were used as negative controls. The following day, LSECs, Kupffer cells, HSCs and hepatocytes were isolated and cultured. Cells were then lifted from the plate with trypsin 10×, or 1× for hepatocytes, for 5–10 min at 37 °C, collected and centrifuged at 800× *g* for 5 min. The pellet was resuspended in PBS at 5% FBS with DAPI (1:1000 dilution), or propidium iodide (1:50 dilution) in the case of HSCs, and cells were analyzed by means of flow cytometry. Results were obtained from three or five animals for each of the study groups, with three determinations per sample.

### 2.13. Determination of Simvastatin in Muscle Tissue

A single dose of oral (inactive) or intravenous (active) simvastatin, and PM-Sim, FPM-CD32b-Sim, FPM-CD36-Sim or FPM-CD32b-CD36-Sim was administered at 20 mg/kg to healthy rats for simvastatin detection in muscle after 10 h of treatment (*n* = 3 animals/group). A sample of ground quadriceps femoris was dissolved in methanol/distilled water (1:1 volume, final concentration 0.2 g muscle/mL) and sonicated (3 × 15 s). The homogenate was centrifuged (13,000× *g*, 10 min, 4 °C) and the supernatant was processed for simvastatin extraction: 50 μL of muscle homogenate was mixed with 125 μL of acetonitrile and vortexed for 30 s. Then, 25 μL of 5 M ammonium formate (NH_4_HCO_2_) buffer (pH 4) was added and the mixture was vortexed again. The sample was centrifuged (13,000× *g*, 10 min, 4 °C) and the supernatant was analyzed via UPLC-MS/MS (Xevo TQ Absolute) for the quantification of active simvastatin.

### 2.14. Maximum Tolerated Dose

To study the safety profile of simvastatin-loaded FPMs, a three-phase maximum tolerated dose assay was conducted, in which acute toxicity (phase 1 and phase 2) and subacute toxicity (phase 3) of treatment with the FPM-CD36-Sim formulation (referred to as FPM-Sim) were evaluated. After each phase, serum and liver tissue samples were obtained from the animals for biochemical and histological analysis, respectively. All phases were performed in healthy mice, and the weight and condition of the animals were monitored throughout the process.

#### 2.14.1. Acute Toxicity: Phase 1 and Phase 2

In phase 1, different doses of encapsulated simvastatin (10, 20 and 50 mg/kg) were tested by administering a single intravenous dose and drawing samples at different times (4 h, 48 h and 1 week) post-treatment (*n* = 2 animals/group). Untreated animals were used as the control group. The dose that showed no toxicity or mortality (FPM-CD36-Sim 10 mg/kg) was further evaluated in phase 2, where it was given intravenously 3 days/week for 2 weeks to monitor the cumulative effect of the drug on the animals compared to the control condition (*n* = 5 animals/group).

#### 2.14.2. Subacute Toxicity: Phase 3

In the subacute toxicity phase, FPM-CD36-Sim 10 mg/kg was administered intravenously 5 days/week for 3 weeks, simulating a longer treatment situation. For this phase, a vehicle group receiving intravenous saline injections was conducted as a control (*n* = 10 animals/group).

### 2.15. Animal Treatment

In order to assess the efficacy of FPM-Sim in BDL rats, animals were randomly assigned to each of the following study groups: control (untreated) (*n* = 9), oral simvastatin 10 mg/kg (*n* = 15), PM-Sim 5 mg/kg (*n* = 9), FPM-CD32b-Sim 5 mg/kg (*n* = 9), FPM-CD36-Sim 5 mg/kg (*n* = 14) and FPM-CD32b-CD36-Sim 5 mg/kg (*n* = 10). Rats were treated from day 22 of BDL and administered daily for 7 days.

In the case of the TAA model, animals were randomly distributed in each study group as follows: control (untreated) (*n* = 9), oral simvastatin 10 mg/kg (*n* = 10), PM-Sim 5 mg/kg (*n* = 10), FPM-CD32b-Sim 5 mg/kg (*n* = 10), FPM-CD36-Sim 5 mg/kg (*n* = 10) and FPM-CD32b-CD36-Sim 5 mg/kg (*n* = 10). Rats were treated during the last 2 weeks of the 8-week model generation, receiving 5 doses/week (10 total doses).

### 2.16. Hemodynamic Measurements

The measurement of hemodynamic parameters was performed in fasted conditions (O/*n*) 90 min after the last treatment administration. Mean arterial pressure (MAP; mmHg) was measured by means of catheterization of the femoral artery and portal pressure (PP; mmHg) via ileocolic vein catheterization using highly sensitive pressure transducers from a PowerLab data acquisition device associated with the physiological data analysis software LabChart 5.0 (ADInstruments, Dunedin, New Zealand). Superior mesenteric artery (SMA) blood flow (SMABF; (mL/min)·100 g) and portal blood flow (PBF; (mL/min)·100 g) were evaluated with a 1.0 mm-diameter ultrasonic perivascular flowprobe connected to a TS420 Perivascular Flow Module (Transonic Systems Inc., Ithaca, NY, USA). SMA resistance (SMAR; (mmHg·min)/(mL·100 g)) and IHVR ((mmHg·min)/(mL·100 g)) were calculated as ((MAP-PP)/SMABF) and (PP/PBF), respectively. Once the hemodynamic study was completed, blood was collected for biochemistry, and liver tissue samples were collected for histological and molecular analysis.

### 2.17. Biochemical Analysis

Fasting blood and serum samples were analyzed to determine creatinine, total bilirubin, alanine aminotransferase (AST), aspartate aminotransferase (ALT), alkaline phosphatase (ALP), creatine kinase (CK), total cholesterol, triglycerides and albumin values. Samples were measured in the automatized CORE laboratory from Hospital Vall d’Hebron, by means of the analytical platform ATELLICA Solution, using Standard CE Mark-approved IVDR diagnostic kits provided by Siemens Healthineers (Erlangen, Germany).

### 2.18. Sirius Red Staining

Liver samples were fixed in 4% paraformaldehyde, embedded in liquid paraffin at 65 °C, and sectioned in 4 μm-thick slices. Once hydrated, samples were dried and stained with 0.1% Picro-Sirius red for 1 h at RT under gentle agitation. Samples were then mounted with DPX rapid mounting medium (Panreac Química SLU, Castellar del Vallès, Spain).

### 2.19. Immunohistochemistry

Immunohistochemistry detection of CD32b receptor was carried out on frozen OCT-embedded 8 µm sections of rat liver. Sections were incubated with primary antibody against SE-1 (Cd32) at 1/100 dilution (NB110–68095, Novus Biologicals, Centennial, CO, USA), followed by EnVision + Dual Link System-HRP (Dako, Glostrup, Denmark) and visualized with the VIP substrate kit (Vector, Burlingame, CA, USA). Samples were counterstained with hematoxylin. Quantitative analysis of stained areas was carried out with Image J software version 1.54d (http://imagej.org (accessed on 13 September 2023)) [28] on ten fields per sample, randomly captured at 10× magnification with an optical microscope Olympus BX61 (Olympus, Hamburg, Germany).

### 2.20. Gene Expression Analysis

Healthy LSECs were treated O/N (37 °C, 5% CO_2_) with free activated simvastatin or PM/FPM-Sim at a drug concentration of 2.5 μM. Control wells were treated with empty (Ø) micelles. Liver samples from treated rats were collected in RNA*later* stabilization Solution (Invitrogen, Thermo Fisher Scientific, USA) and kept for 1 week at 4 °C. Total RNA was extracted from liver cells or tissue and converted to cDNA. From each sample, 20 ng of cDNA was amplified with specific TaqMan probes for COL1A1 (collagen type I alpha 1 chain; Rn 01463848_m1) and KLF2 (Rn 01420496_gH). The relative gene expression was normalized to GAPDH (glyceraldehyde-3-phosphate dehydrogenase; Rn 99999916_s1). Each sample was analyzed in triplicate.

### 2.21. Western Blot

Rat liver samples containing 60 µg of protein were run on a NuPAGE Bis-Tris 4–12% SDS-PAGE (Invitrogen, Thermo Fisher Scientific, USA) under denaturing conditions, blotted onto a polyvinylidene difluoride (PVDF) blotting membrane and incubated with the appropriate dilution of the primary antibody for eNOS (1:500 in TTBS 1X; BD, USA), p-eNOS (1:250 in 5% BSA; Cell Signaling Technology, Danvers, MA, USA) and KLF2 (1:200 in 5% BSA; Santa Cruz Biotechnology, Santa Cruz, CA, USA). GAPDH antibody (1:5000 in TTBS 1X, Ambion, Austin, TX, USA) was used as the loading control. The electrophoretic bands of Western blots were detected through digital chemiluminescence development using an Odyssey Fc Imaging System (LI-COR Biosciences, Lincoln, NE, USA). Protein quantification was performed with Image Studio Lite software version 5.2 (LI-COR Biosciences, USA).

### 2.22. Statistical Analysis

IBM SPSS Statistics 20 (IBM, Armonk, NY, USA) was used for statistical analysis. Quantitative results were expressed as mean ± standard error of the mean (SEM) and compared with analysis of variance followed by unpaired Student’s *t* test (between two groups) or one-way analysis of variance (ANOVA) with Tukey’s HSD post hoc correction (among three or more groups). When data were not normally distributed, nonparametric tests were applied, using the Mann–Whitney U test to compare two groups and the Kruskal–Wallis test for multiple comparisons. Pearson’s correlation coefficient was calculated to show the correlation of two parameters. A *p*-value ≤ 0.05 was considered statistically significant.

## 3. Results

### 3.1. FPM Physicochemical Characterization

**Figure 1:**
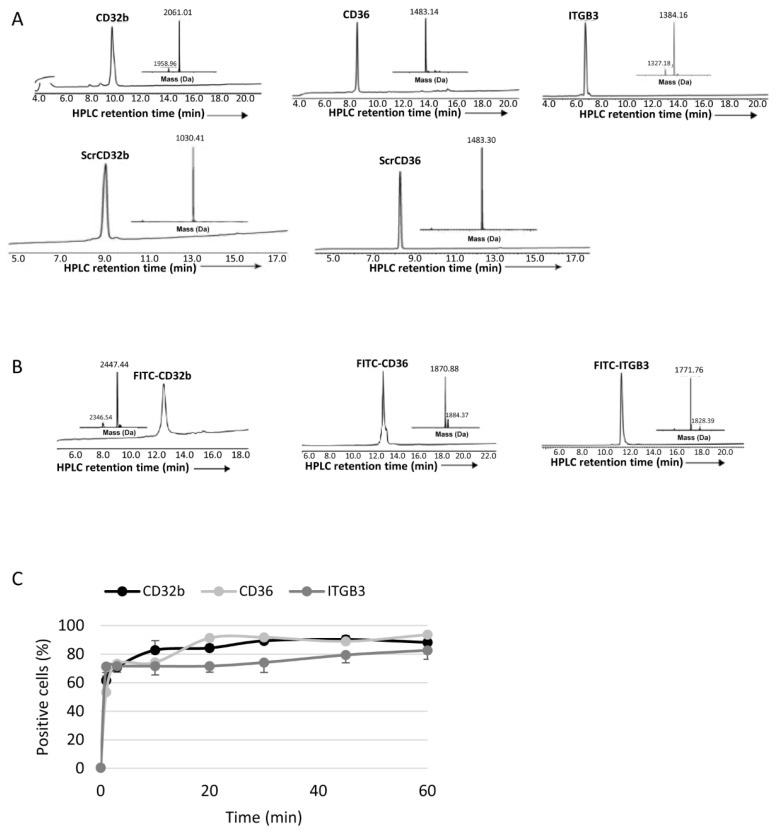
Characterization of peptide ligands. (**A**) RP-HPLC chromatogram analysis of unlabelled specific and the scrambled variants: CD32b, CD36, ITGB3, ScrCD32b and ScrCD36. (**B**) RP-HPLC chromatogram analysis of FITC-labelled peptides: FITC-CD32b, FITC-CD36 and FITC-ITGB3. (**C**) In vitro internalization of labelled peptide ligands analysed via flow cytometry after treating LSECs isolated from healthy rats at different times (from 1 to 60 min). Results are plotted as mean ± SEM. *n* = 2 animals per experimental condition.

CD32b, CD36 and ITGB3 peptide ligands, with or without fluorescein isothiocyanate (FITC) labelling, and the scrambled versions of CD32b and CD36 peptide ligands were synthesized (Figure 1A,B) via Fmoc-solid phase peptide synthesis (Fmoc-SPPS) using standard methods. Internalization of FITC-labelled peptides in healthy LSECs showed a high in vitro cellular uptake, with more than 50% of LSECs internalizing the three ligands after 1 min of incubation and, at 1 h, positive cells were higher than 80% in all cases (Figure 1C).

**Figure 2:**
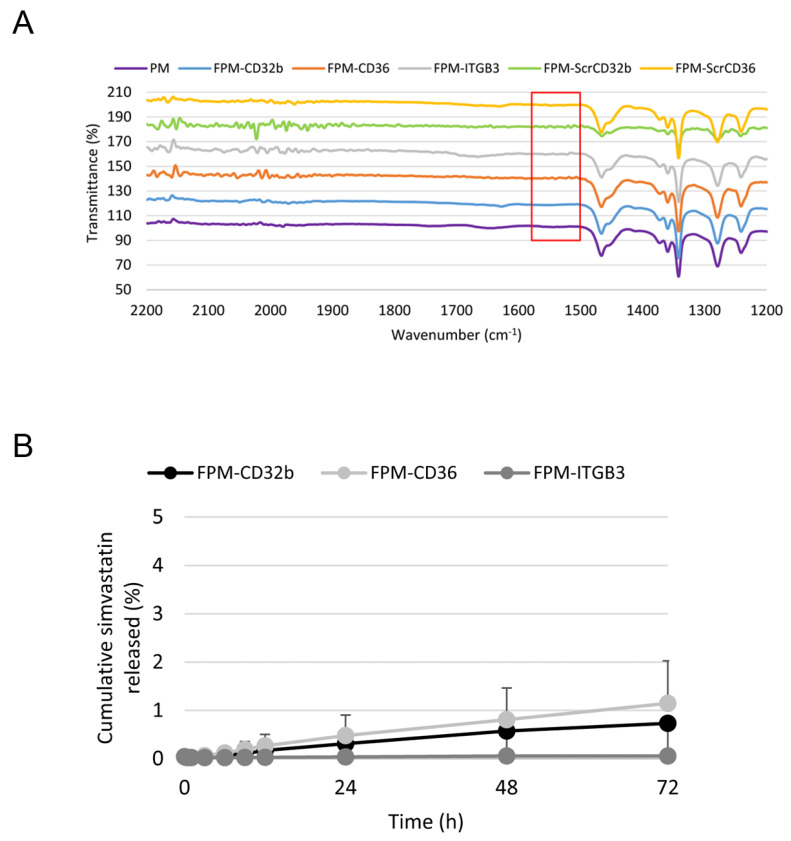
Characterization of functionalized polymeric micelles. (**A**) FTIR spectra of PMs and FPMs with the different peptide ligands (specific and scrambled). The red square indicates the peaks generated by −COOH groups from the modified polymer and −NH^2^ from bound peptides. (**B**) In vitro drug release kinetics under physiological conditions. Data are expressed as mean ± SEM. *n* = 2 per group.

Fourier-transform infrared spectroscopy (FTIR-spectra) demonstrating the correct coupling of the peptide ligands to PMs is shown in Figure 2A.

The physicochemical characteristics of FPMs are summarized in Table 1. The mean hydrodynamic diameter of FPMs varied according to the ligand used for functionalization, ranging from 180 to 290 nm. The observed nanoparticle size is due to an increase in functional groups and charges from the carboxylate side of the polymer and peptide, attracting water molecules to the surface of the PMs, as the hydrodynamic diameter is the sum of the geometric size plus the layer of water molecules on the surface of the particle. Digital image analysis performed based on transmission electron microscopy photographs demonstrated a much smaller mean diameter (≈20 nm), difficult to eliminate via the reticuloendothelial system. All formulations had mid-range polydispersity index values (ranging from 0.35 to 0.53) and presented a zeta potential close to neutrality, being positive for all nanoparticles except FPM-ITGB3. Finally, the encapsulation efficacy of active simvastatin (20 mg/mL) within FPMs (100 mg/mL Pluronic F127:1 mg/mL peptide) (FPM-Sim) was greater than 95% for all formulations. The stability of FPM-Sim was confirmed by the cumulative percentage of the drug released in a medium simulating physiological condition (at 37 °C and pH 7.4) being below 1.5% in all types of functionalized micelles, for at least 72 h (Figure 2B).

### 3.2. FPM In Vitro Internalization in LSECs

**Figure 3:**
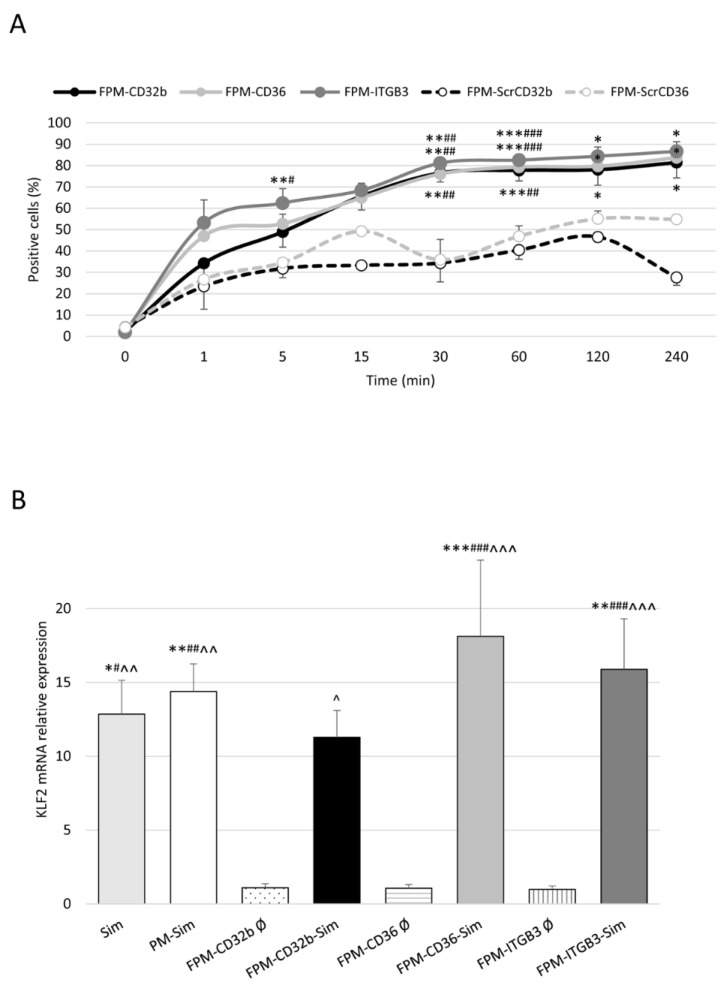
In vitro internalization and functionality of functionalized polymeric micelles. (**A**) Internalization of FPMs in healthy LSECs evaluated via flow cytometry from 1 min to 4 h. Values are represented as mean ± SEM. *n* = 3 animals for each formulation. * *p* ≤ 0.05, ** 0.01, *** 0.001 vs. FPM-ScrCD32b; # *p* ≤ 0.05, ## 0.01, ### 0.001 vs. FPM-ScrCD36. (**B**) Relative quantification of KLF2 mRNA expression via qRT-PCR in LSECs isolated from healthy rats. GAPDH was used as endogenous control and results were normalized to the average expression of the empty nanoparticles functionalized with the three different ligands. mRNA levels are expressed as mean ± SEM. *n* = 3 animals per experimental condition. * *p* ≤ 0.05, ** 0.01, *** 0.001 vs. FPM-CD32b empty; # *p* ≤ 0.05, ## 0.01, ### 0.001 vs. FPM-CD36 empty; ^ *p* ≤ 0.05, ^^ 0.01, ^^^ 0.001 vs. FPM-ITGB3 empty.

Flow cytometry was used to measure the in vitro internalization of FPMs, labelled with 5-DTAF (5-(4,6-dichlorotriazinyl) aminofluorescein), in LSECs isolated from healthy rats. Cell uptake was found to be more efficient for nanoparticles functionalized with the specific ligands for CD32b, CD36 and ITGB3 than those functionalized with the scrambled versions ScrCD32b and ScrCD36, demonstrating the importance of specific recognition for correct targeting. Figure 3A shows that after 5 min of treatment, a difference in uptake between the two types of functionalization was evident. This difference became significant at 30 min and, at 4 h, the internalization of specific FPMs was over 80% (FPM-CD32b: 81%, FPM-CD36: 84%, FPM-ITGB3: 87%), whereas the scrambled FPMs were only internalized in 55% (FPM-ScrCD36) and 28% (FPM-ScrCD32b) of the total LSECs.

### 3.3. FPM In Vitro Functionality in LSECs

The functional effect of simvastatin was measured by following the expression of KLF2 in isolated primary LSECs treated with simvastatin, either encapsulated (PMs and FPMs) or in its free form. In its encapsulated form, FPM-Sim was equally as effective as free simvastatin or PM-Sim, causing a significant overexpression of KLF2 in healthy LSECs (Figure 3B). In contrast, no changes in KLF2 expression were observed when LSECs were treated with empty nanoparticles, ruling out any possible functional effect of the empty nanodevices.

### 3.4. FPM In Vivo Internalization in Liver Cells

**Figure 4:**
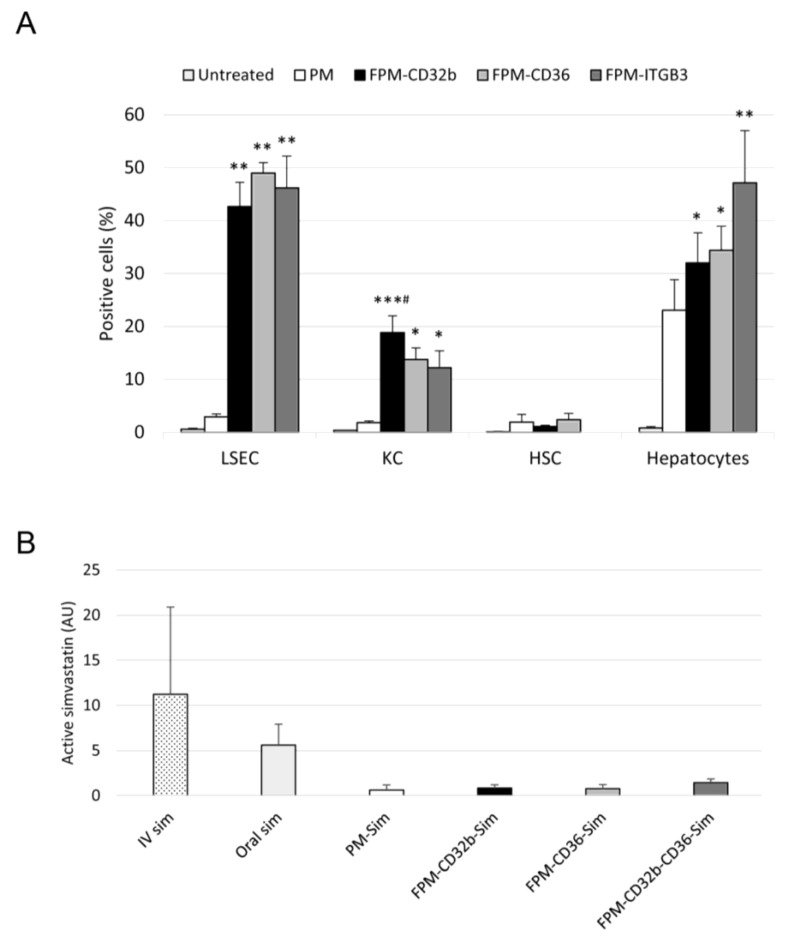
In vivo internalization of FPMs in liver cells and simvastatin quantitation in muscle. (**A**) Internalization of nanoparticles in different hepatic cell types (LSECs, Kupffer cells, HSCs and hepatocytes) after intravenous treatment of healthy rats with PMs or FPMs. Results are represented as mean ± SEM. *n* = 3–5 animals per condition. * *p* ≤ 0.05, ** 0.01, *** 0.001 vs. untreated; # *p* ≤ 0.05 vs. PMs. (**B**) Area of active simvastatin determined by means of UPLC-MS/MS in muscle samples of healthy rats treated with free simvastatin (oral or intravenous) or encapsulated simvastatin (PMs or FPMs). Results are represented as mean ± SEM. *n* = 3 animals per condition.

Healthy rats were treated with the three 5-DTAF-labelled FPMs and the quantitation of internalization in the four main types of hepatic cells was carried out by means of flow cytometry and compared with non-functionalized PMs (Figure 4A). The binding of specific peptide ligands on the surface of polymeric micelles led to an increase in the delivery of these nanoparticles to LSECs by more than 13-fold (FPM-CD32b: 42.64%, FPM-CD36: 49%, FPM-ITGB3: 46%) compared to non-functionalized ones (3%). In Kupffer cells, there was also a significantly higher percentage of positive cells for FPM-CD32b compared with PMs (*p* = 0.041), but the internalization rate of CD32b micelles only reached 19% in liver resident macrophages. The other two FPM formulations also produced increases in cellular uptake in Kupffer cells, but in a more discreet manner. By contrast, HSCs displayed virtually no internalization of any kind of nanoparticle after in vivo treatment. Finally, hepatocytes showed a number of internalized FPM-ITGB3 and PMs, with 47% and 23% of cells being positive, respectively. Due to this observed undesired high internalization of FPM-ITGB3 in hepatocytes, we decided to discard this formulation for the following in vivo studies, selecting CD32b and CD36 peptide ligands either alone (FPM-CD32b or FPM-CD36) or in combination in a mixed functionalization (FPM-CD32b-CD36).

### 3.5. Simvastatin Content in Muscle after In Vivo Administration

Considering that muscle toxicity is known to be the main adverse effect of oral simvastatin, the presence of active simvastatin in muscle from healthy treated rats was quantified via UPLC-MS/MS (ultra-high-performance liquid chromatography coupled to tandem mass spectrometry), comparing the administration of free (oral and intravenous) and encapsulated formulations (PMs and FPMs). Higher amounts of active simvastatin in muscle were observed in the group of animals treated intravenously, followed by those receiving oral simvastatin, compared with the very low values obtained when simvastatin was loaded in PMs and FPMs (Figure 4B).

### 3.6. Safety and Toxicity Assay

**Figure 5:**
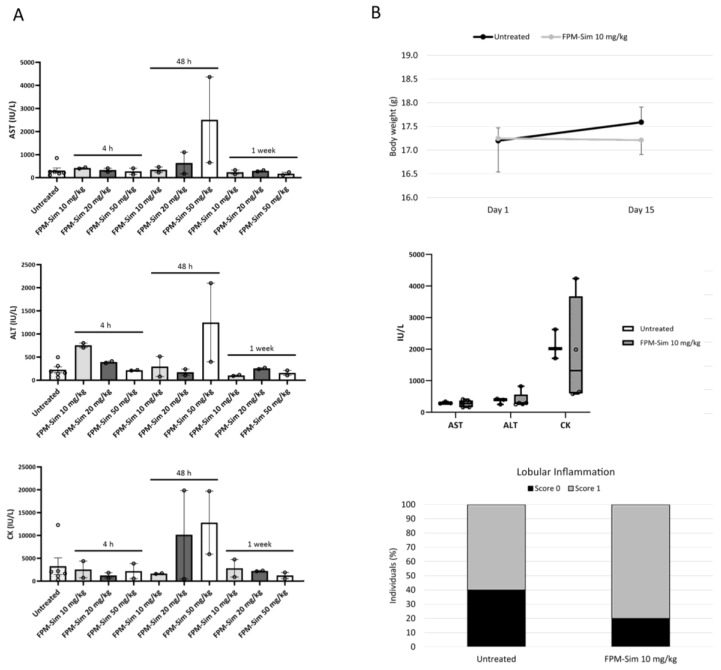
Acute toxicity studies (phases 1 and 2) of the maximum tolerated dose assay (**A**) Phase 1: Serum AST, ALT and CK levels of untreated healthy mice and after a single dose of FPM-CD36-Sim at 10, 20 or 50 mg/kg analyzed at 4 h, 48 h and 1 week. Values are plotted as mean ± SEM. *n* = 2 per group. (**B**) Phase 2: Evolution of body weight (up) of untreated healthy mice and after treating with intravenous FPM-CD36-Sim 10 mg/kg for 3 days/week for 2 weeks; serum AST, ALT and CK levels at the end of the study (values expressed in box plots; *n* = 5 for each condition) (center) and percentage of individuals with lobular inflammation (bottom).

The maximum tolerated dose was determined in healthy mice in a three-phase study using FPM-CD36 as a reference nanoparticle to assess the toxicity of simvastatin-loaded FPMs. The first and the second phases of the study corresponding to acute protocols (Figure 5A,B) allowed us to establish a well-tolerated dose of encapsulated simvastatin at 10 mg/kg to avoid temporary elevations of AST, ALT and CK in some individuals.

**Figure 6:**
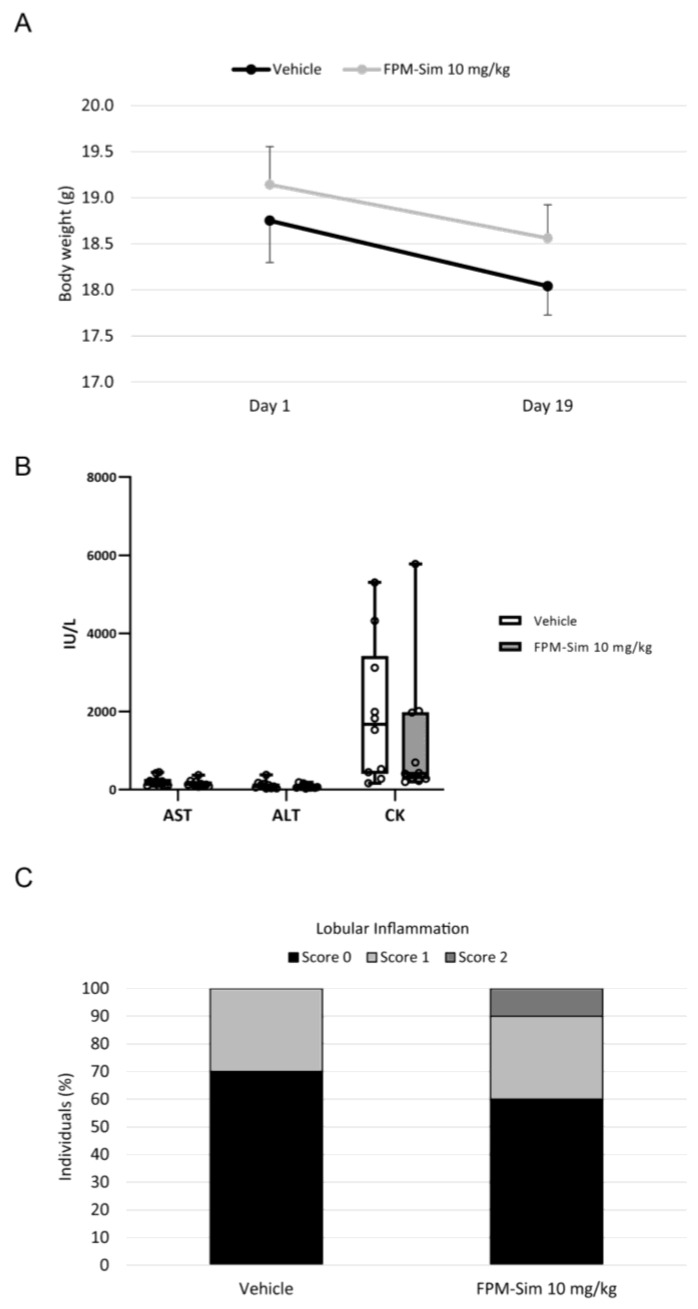
Sub-acute toxicity study (phase 3) of the maximum tolerated dose assay. (**A**) Change in body weight of healthy mice treated intravenously with vehicle (saline) or FPM-Sim 10 mg/kg for 5 days/week for 3 weeks. (**B**) Serum AST, ALT and CK levels after treatment. Values are expressed in box plots. *n* = 10 for each experimental condition. (**C**) Scoring of lobular inflammation.

Finally, a third subacute protocol was performed, where mice were treated with FPM-CD36-Sim 10 mg/kg for 5 days/week for 3 weeks. A group of control animals also received intravenous injections of saline (vehicle group) to normalize undesired effects of animal handling. Animal behavior was not altered in any aspect, but both vehicle and treated mice experienced a similar weight loss during the 3 weeks of the study, probably due to the manipulation associated with intravenous injection (Figure 6A). Biochemical parameter analysis revealed no signs of liver or muscle toxicity in animals treated with FPMs (Figure 6B). The histological analysis showed that only one animal receiving encapsulated simvastatin showed a higher score of lobular inflammation than the individuals in the vehicle group (Figure 6C).

All subsequent in vivo experiments were performed with a 10 mg/kg dose of simvastatin-loaded FPMs.

### 3.7. FPM Effectivity in an Advanced Model of Cirrhosis (BDL)

**Figure 7:**
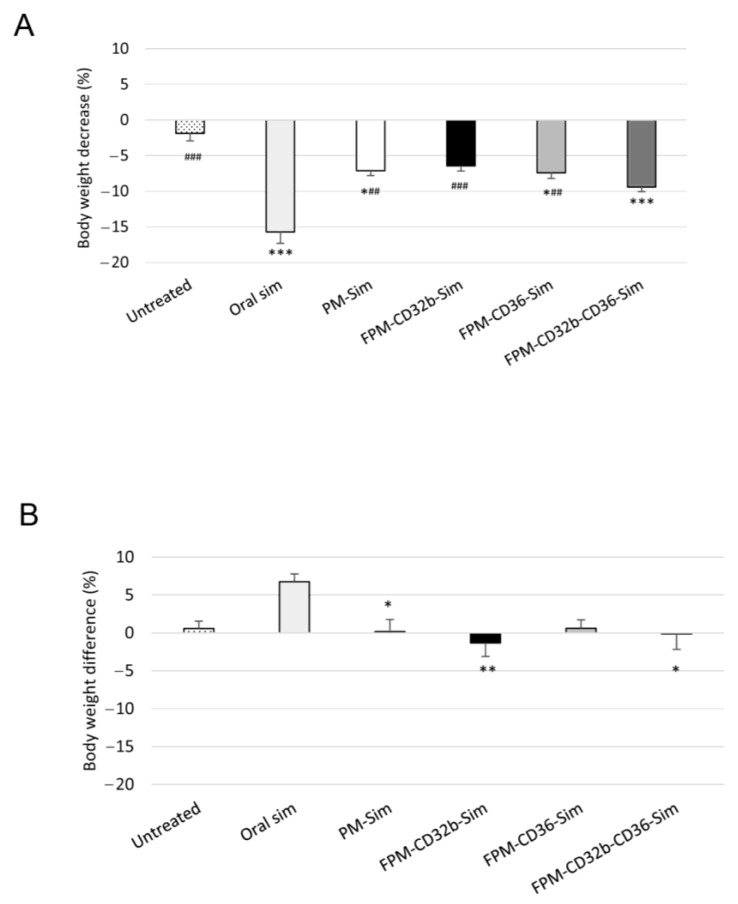
Body weight changes during treatment. (**A**) In the experimental BDL model, body weight decreases in untreated (*n* = 9) and treated rats after 1 week of daily treatment with oral simvastatin 10 mg/kg (*n* = 13), PM-Sim 5 mg/kg (*n* = 9), FPM-CD32b-Sim 5 mg/kg (*n* = 9), FPM-CD36-Sim 5 mg/kg (*n* = 12) or FPM-CD32b-CD36-Sim 5 mg/kg (*n* = 9). (**B**) Body weight differences in the experimental model of TAA-induced cirrhosis, in untreated (*n* = 9) and treated rats after 2 weeks administration (5 days/week) of oral simvastatin 10 mg/kg (*n* = 10), PM-Sim 5 mg/kg (*n* = 10), FPM-CD32b-Sim 5 mg/kg (*n* = 10), FPM-CD36-Sim 5 mg/kg (*n* = 10) or FPM-CD32b-CD36-Sim 5 mg/kg (*n* = 10). Data are represented in box plots. * *p* ≤ 0.05, ** *p* ≤ 0.01, *** *p* ≤ 0.001 vs. untreated; ## *p* ≤ 0.01, ### *p* ≤ 0.001 vs. oral simvastatin.

In the BDL model, mimicking decompensated cirrhosis, the efficacy of FPM-Sim compared to PM-Sim and oral simvastatin was evaluated after 1 week of daily treatment. After the administration of seven doses, oral simvastatin caused the most significant body weight decrease compared with untreated BDL animals. This reduction was also greater than the moderate weight loss observed in all groups receiving encapsulated simvastatin, despite the stress caused by animal manipulation during the injection of PMs or FPMs (Figure 7A).

At the biochemical level, oral administration of simvastatin induced the highest values of AST and ALT, and of CK (Supplementary Table S1). On the other hand, in this advanced cirrhotic model, the use of nanoparticles generated a significant increase in triglycerides and a discrete increase in total cholesterol when compared with untreated BDL rats or treated with oral simvastatin (Supplementary Table S1).

The hemodynamic studies showed that animals treated orally with simvastatin presented a significant reduction in PP when compared with the untreated control group, with a reduction of 3.92 mmHg (*p* = 0.004), and with respect to PM-Sim (*p* = 0.037) (Table 2). A reduction in PP was also observed in the animals treated with functionalized micelles FPM-CD32b-Sim and mixed-FPM-Sim. The rest of the hemodynamic parameters studied were not affected.

### 3.8. FPM Effectivity in a Non-Decompensated Model of Cirrhosis (TAA)

A less severe liver disease model (8-week TAA model) was generated, with the rats receiving treatment 5 days/week during the last 2 weeks of the model. Simvastatin did not cause animal weight loss, except for a slight reduction in body weight of less than 2% in the FPM-CD32b and mixed-FPM treatment groups, most probably associated with the stress induced by intravenous administration (Figure 7B). Compared with untreated BDL rats, a marked overall decrease in liver transaminase levels was observed in untreated TAA individuals, as well as in cholesterol and triglycerides. However, there was still a significant elevation of triglycerides in TAA groups when treating with polymeric micelles (Supplementary Table S2).

On the other hand, as shown in Table 3, treatment with FPM-CD32b-Sim significantly decreased PP by more than 2 mmHg compared with the untreated control group (*p* = 0.005), and with respect to oral simvastatin (*p* = 0.037) and FPM-CD36-Sim (*p* = 0.014). Also, FPM-CD32b-CD36-Sim decreased PP levels in relation the untreated group (*p* = 0.042). In addition, a slight but consistent decrease in IHVR and SMAR in all groups treated with encapsulated simvastatin formulations suggests an overall improvement in portal hypertension.

**Figure 8:**
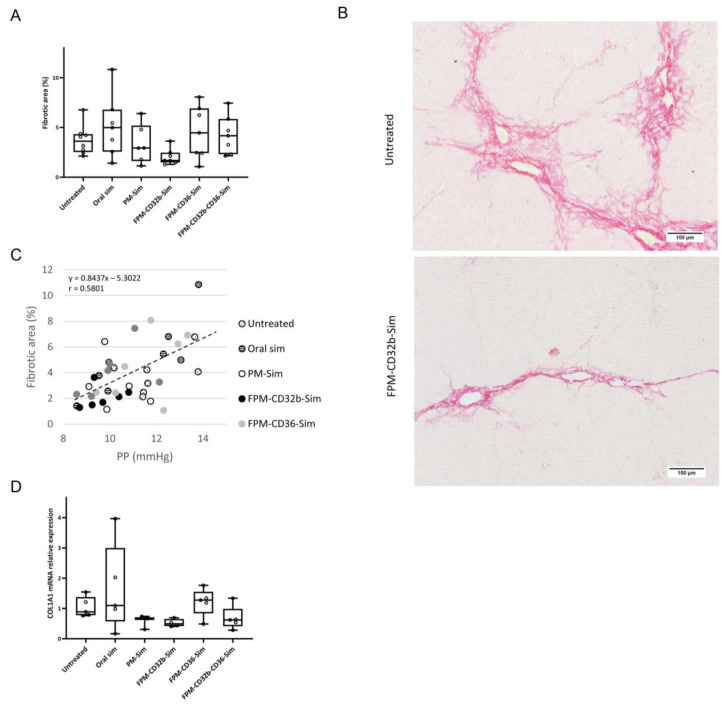
Efficacy of functionalized polymeric micelles in an experimental model of thioacetamide-induced cirrhosis. (**A**) Percentage of fibrotic area in the liver of untreated (*n* = 7) and treated rats after 2 weeks of 5 days/week treatment with oral simvastatin 10 mg/kg (*n* = 7), PM-Sim 5 mg/kg (*n* = 6), FPM-CD32b-Sim 5 mg/kg (*n* = 7), FPM-CD36-Sim 5 mg/kg (*n* = 7) or FPM-CD32b-CD36-Sim 5 mg/kg (*n* = 6). Values are presented in box plots. (**B**) Representative images of Sirius Red staining at 10× magnification in liver sections. (**C**) Correlation between portal pressure values and percentage of fibrotic area. The line represents the linear trend. The equation and the r value are displayed on the graph. (**D**) Relative quantification of COL1A1 mRNA expression via qRT-PCR in total liver samples. GAPDH was used as the endogenous control and results were normalized to the untreated group. mRNA levels are represented in box plots. *n* = 5 per group.

To elucidate the possible causes of this improvement in portal hemodynamics, the detection of collagen fibers via Sirius red staining was performed in liver samples from these animals. Figure 8A depicts the quantitation of the fibrotic area in all groups, showing that TAA rats treated with the FPM-CD32b-Sim formulation presented the lowest percentage (2.05%) compared with the other groups; this result is consistent with the improvement in PP caused by nanoparticles functionalized with CD32b. In this sense, both parameters, PP and fibrotic area, showed a significantly positive correlation (r = 0.580; *p* < 0.001), portraying the relationship between scar tissue formation in the liver, IHVR, and portal hypertension (Figure 8C). Likewise, analysis of gene expression in total liver samples revealed down-regulation of the COL1A1 gene, encoding the major component of type I collagen, when rats received simvastatin encapsulated in FPM-CD32b (Figure 8D).

**Figure 9:**
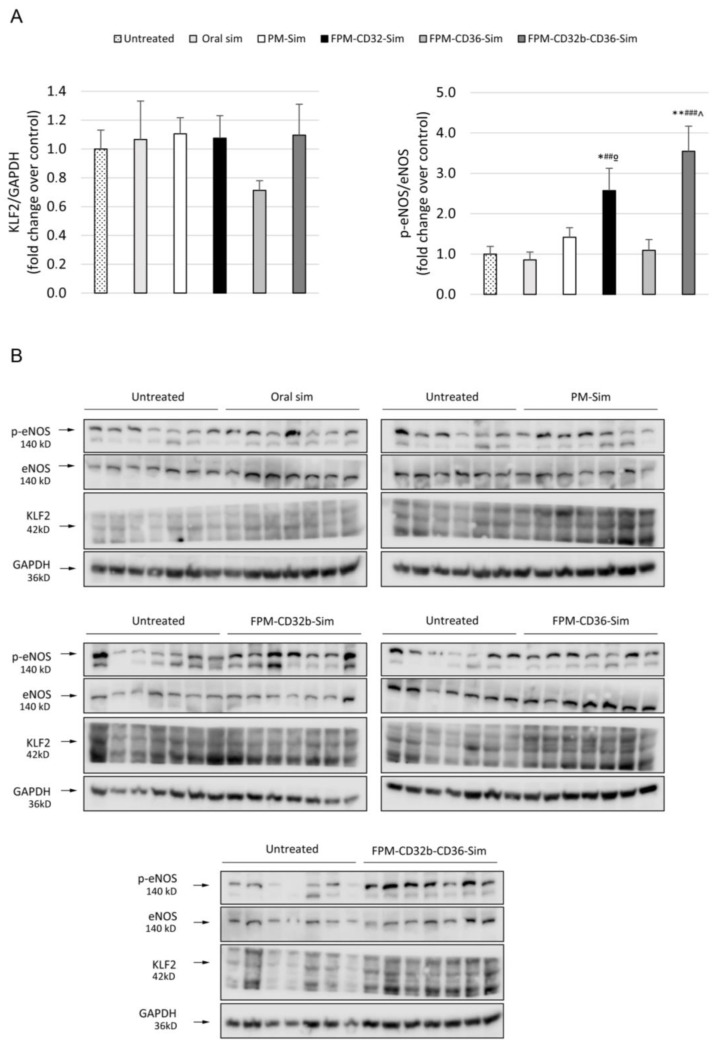
Western blot analysis of intrahepatic endothelial dysfunction markers. (**A**) Quantification of KLF2 and p-eNOS/eNOS protein levels in untreated (*n* = 7) and treated rats after 2 weeks of 5 days/week treatment with oral simvastatin 10 mg/kg (*n* = 7), PM-Sim 5 mg/kg (*n* = 6), FPM-CD32b-Sim 5 mg/kg (*n* = 7), FPM-CD36-Sim 5 mg/kg (*n* = 7) or FPM-CD32b-CD36-Sim 5 mg/kg (*n* = 7). Specific bands for each protein, identified by molecular weight, are indicated with an arrow. Protein levels are represented in box plots. * *p* ≤ 0.05, ** *p* ≤ 0.01 vs. untreated; ## *p* ≤ 0.01, ### *p* ≤ 0.001 vs. oral simvastatin; ^ *p* ≤ 0.05 vs. PM-Sim; º *p* ≤ 0.05, vs. FPM-CD36-Sim. (**B**) Photographs of the complete agarose gels containing p-eNOS, eNOS and KLF2 protein bands are shown. All gels were run with the untreated group for normalization between groups. GAPDH was used as loading control.

Finally, the protein expression levels of endothelial dysfunction markers were assessed in total liver samples, showing that, despite there being no significant difference in KLF2 expression between treated and untreated rats, activation of eNOS was significantly promoted when simvastatin was administered in FPM-CD32b-CD36 and FPM-CD32b, as shown in Figure 9.

### 3.9. CD32b Expression in LSECs from Different Models of Liver Disease

**Figure 10:**
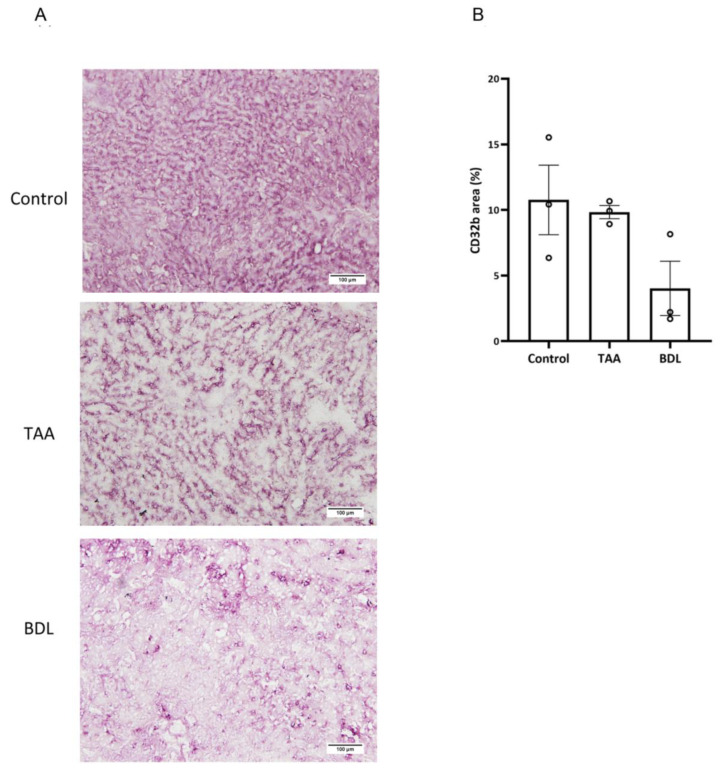
Immunohistochemical assessment of CD32b expression in two models of liver disease. (**A**) Representative images of CD32b immunostaining at 10× magnification in liver sections of healthy control, TAA-induced cirrhosis and BDL rat models. Positive staining is clearly defining the liver sinusoids. (**B**) Bar chart showing immunohistochemical quantitation of CD32b expression levels, represented as mean ± SEM (*n* = 3 per group).

Dedifferenciation of LSECs in liver disease is associated, in the most recent literature, with loss of the CD32b-specific marker [29]. To assess the degree of CD32 expression that still remains in the LSECs obtained from the liver disease models used in this study, the immunohistochemistry of CD32b in frozen sections of liver samples was carried out. Analysis of the CD32b staining area surrounding the liver sinusoids showed that this specific marker is not equally lost in the two models, demonstrating higher expression levels in the TAA model compared with BDL model (Figure 10).

## 4. Discussion

In an attempt to provide a solution for improving the therapeutic window of statins for the treatment of advanced CLD, this work addresses the optimization of a simvastatin delivery system enhancing the targeting of LSECs. The optimization included the design and functionalization with peptide ligands of Pluronic-based PMs loaded with simvastatin. We studied the in vitro characteristics in primary cultures of liver cells, and the in vivo effect in animal models of CLD, demonstrating the increased therapeutic potential of the loaded drug by reducing portal hypertension and liver fibrosis in a non-decompensated CLD animal model.

To increase the accumulation of nanoparticles in LSECs, peptide ligands recognizing three LSEC receptors were designed and synthesized according to various expression criteria (either present in functional, dysfunctional or in both LSEC differentiation stages) and their ability to enter into primary LSECs.

The coupling of these peptides on the surface of PMs generated three FPM formulations with different features, but with homogeneous particle shape and size, and a zeta potential close to neutrality. This was due to the use of polyethylene glycol (PEG) at the hydrophilic end of the polymer, which prevents aggregation between the uncharged nanoparticles [30]. The effect of an encapsulated drug can be maximized and the side-effects of the drug minimized only if a micelle is stable enough to retain most of the drug until the target is reached. Our result indicated that FPMs were thermodynamically stable, since no simvastatin was released until at least 72 h under neutral pH conditions, simulating the blood circulation [31,32].

In vitro internalization in primary cultures of LSECs of the three FPM formulations with the specific ligands for CD32b, CD36 and integrin α_V_β_3_ receptors demonstrated that specific ligand–receptor interactions plus passive targeting promoted uptake by a greater number of healthy LSECs than the micelles conjugated with their scrambled variants.

Simvastatin has been shown to reduce portal hypertension through the putative reduction in IHVR by means of several mechanisms, including the induction of KLF2 expression, related to the stimulation of a vasoprotective phenotype in LSECs [11,12,33]. This effect was confirmed by the expected overexpression of KLF2 in healthy isolated LSECs treated with free or encapsulated simvastatin. We also ruled out any possible effect of the empty functionalized nanodevices acting only as an inert vehicle for the delivery of the loaded drug.

Intravenous treatment of healthy rats with the different fluorescent formulations confirms that, in all cases, functionalization confers greater efficiency in entering the main liver cells than passive targeting; the capture of FPMs was clearly superior to that achieved by non-functionalized PMs, and LSECs were the liver cells with the highest uptake. Furthermore, in endothelial cells, all three types of functionalization were equally effective. In vivo cell internalization experiments also confirmed that the receptors selected as LSEC targets for our nanoparticles were also expressed in healthy Kupffer cells and hepatocytes, allowing functionalized nanoparticles to enter both cell types quite efficiently. This was expected for CD36 and integrin α_V_β_3_, as their expression in the liver is elevated not only in LSECs, but also in other hepatic cell types [34,35]. However, CD32b, an FcγR conferring LSECs the highest endocytic capacity of any cell in the human body, is known to be the most specific marker of these cells in the liver. Yet, recent studies have shown that CD32b is not only expressed in LSECs but also in Kupffer cells, with a liver level expression of 90% and 10%, respectively [36].

The differential accumulation of simvastatin in muscle depending on the different formulations used was a key factor in this study. It is well known that nanoparticles tend to accumulate passively in the liver because this organ is part of the reticuloendothelial system [37,38]. Accordingly, the use of nanoparticle encapsulation decreased the presence of active drug detected in the muscle compared to free simvastatin administered either orally or intravenously. This effect is due to the lower uptake of nanoparticles by skeletal muscle compared with the higher natural retention of nanoparticles in the liver, regardless of their functionalization, since both PMs and FPMs showed an equal reduction in the amount of active simvastatin detected in the muscle.

To determine the dose and schedule of FPM-Sim for the efficacy studies in experimental models of liver diseases, the safety profile was assessed in healthy mice by establishing the maximum tolerated dose in a phased assay, studying the use of different simvastatin doses, as well as different administration patterns. The use of FPM-Sim at high doses of 20 and 50 mg/kg resulted in transient elevations in liver transaminases and CK in some individuals, and these doses were discarded to avoid further toxicity in animal models. In contrast, the 10 mg/kg dose showed no evidence of toxicity in any of the established phases and was scaled in subsequent efficacy studies in rat experimental models of liver disease.

The first efficacy evaluation in an advanced CLD model such as BDL was aimed at improving the PP-lowering effect achieved by PM-encapsulated simvastatin in a previous study [18] using the new functionalized formulations while maintaining the lower levels of toxicity compared with free simvastatin. Encapsulated simvastatin resulted in significantly less body weight reduction at the end of treatment in cirrhotic animals compared to free simvastatin, and lower levels of hepatic and muscular toxicity markers. On the other hand, the use of nanoparticles triggered a significant increase in serum triglycerides and, in a more moderate way, in total cholesterol. This may be explained by the fact that the PM is made from Pluronic F127 (or polaxamer P407), which has been shown to induce a transient hyperlipidemic effect caused by a temporary reduction in the number of fenestrae in LSECs in a dose-dependent manner [39]. Consequently, given that BDL animals at baseline have an advanced capillarized endothelium and a marked reduction in fenestrae due to the endothelial dysfunction induced by bile accumulation [40], the addition of an extra element that closes the fenestrae, even temporarily, may lead to an impaired transendothelial transfer of lipoproteins from sinusoidal blood to the extracellular space of Disse.

In terms of efficacy, oral simvastatin, although causing greater toxicity, was the most effective treatment in significantly reducing PP compared to the untreated control group, while the functionalized nanoparticles FPM-CD32b and mixed-FPM only caused a discrete reduction in the effect of simvastatin on PP compared to the non-functionalized PMs. It is worth mentioning that the BDL model used in the present study, mimicking an advanced CLD, resulted in severely affected animals with unusually high levels of PP in the control group and increased liver transaminase values.

We then performed a second in vivo efficacy study in a model of cirrhosis induced by TAA, a widely used model for its high reproducibility and homogeneity of results, its low mortality, and a lower systemic toxicity [41]. Moreover, the model was developed only for 8 weeks, yielding cirrhotic animals but in a non-decompensated stage of the disease. Simvastatin was administered in different formulations during the last 2 weeks of model generation. In this non-decompensated model, simvastatin induced lower toxicity in all aspects: the body weight of animals was not reduced, and the slight decrease in body weight observed in the groups that received intravenous treatment might be associated with the stress caused by the administration route rather than the drug itself. Furthermore, transaminases and CK values were also diminished in all TAA animals compared to BDL. In addition, the increase in total cholesterol and triglycerides observed in BDL rats was of lower magnitude, which could be explained by the lesser degree of capillarization expected from a more moderate CLD model represented by the 8-week TAA animals.

The reduced toxicity observed in this model was accompanied by a lower PP in the untreated control group compared with the previous BDL model. Treatment with encapsulated simvastatin further reduced PP, with a significant difference in the FPM-CD32b and mixed-FPM formulations. Moreover, the PP-lowering effect achieved by FPM-CD32b-Sim was also significant versus FPM-CD36-Sim and oral free simvastatin. To elucidate this reduction effect, we evaluated the correlation between PP and fibrosis, confirming that the greater the area of liver fibrosis, the greater the PP increase, and vice versa [3,42]. Collagen expression followed the same trend, and in the case of FPM-CD32b-Sim treatment, it was reduced to half compared to the untreated TAA group. Quantification of proteins involved in signaling pathways related to vasoprotection induced by statins administration [14,43,44] showed unaltered expression of KLF2 between treated and untreated animals, suggesting that the dose used in in vivo treatments is not enough to increase the values of this transcription factor. Nonetheless, in line with the improvement in PP reduction, the p-eNOS/eNOS ratio was significantly elevated by FPM-CD32b-Sim and mixed-FPM-Sim, suggesting an amelioration of endothelial dysfunction by stimulating nitric oxide production through the targeted release of simvastatin into LSECs of TAA-induced cirrhotic rats.

We interpret the greater efficacy demonstrated by the FPM-CD32b-Sim nanoparticles in the TAA model as a consequence of enhanced targeting by the specific peptide ligand due to higher levels of CD32b expression in LSECs from this model compared with the BDL model. In this regard, immunohistochemical staining to identify the CD32b antigen in liver sections of rats from both experimental models was significantly higher in TAA than in BDL rats. This is also supported by a recent study conducted in our laboratory [45], in which the percentage of CD32b^+^ LSECs was estimated by means of cell sorting, establishing that the loss of CD32b during the capillarization process might display different features depending on the etiology, disease stage and mechanisms causing liver injury.

One limitation of this work was performing the efficacy studies only in male animals. In our study, we wanted to evaluate as a proof of concept the FPM efficiency by means of the ability to reduce portal pressure in cirrhotic individuals. The vast majority of published studies on the hemodynamic disturbances occurring in liver disease use male rats or mice, because female estrogen hormones modify hemodynamic parameters, increasing or decreasing their values randomly and unrelated to liver disease [22]. However, we acknowledge that to have a complete picture of the efficacy demonstrated by the FPM-CD32b-Sim nanoparticles, further studies are needed using both sexes.

In summary, there are three important observations in this study. First, the adequate functionalization of nanoparticles may provide a noticeable positive effect for passive targeting. Second, it seems clear that, even though the specific targeting of LSECs can be lost gradually in pathological situations, it is more effective to choose specificity over other alternatives where the target may be abundant but ubiquitously expressed. Third, the stage of the disease is a key factor in the use of PM nanoparticles in liver diseases. Our results indicate that in advanced decompensated models, nanoparticles are able to decrease the toxicity caused by simvastatin but, on the other hand, their effectiveness is limited. By contrast, in a less severe stage of the disease, simvastatin encapsulation increases the nanoparticles’ hepatic beneficial impact and minimizes the possible secondary effect of the poloxamer device.

In conclusion, active targeting of a Pluronic-based nanodevice by means of functionalization with peptide ligands for the specific administration of simvastatin towards LSECs reduces muscle and liver toxicity, but without a clear portal pressure reduction, in a decompensated model of cirrhosis. However, in a non-decompensated model of CLD, functionalization with CD32b ligand enhances the efficacy of simvastatin, reducing PP values as well reducing liver fibrosis. This conclusion favors the use of CD32b-functionalized PMs as potential nanotransporters for vasoprotective drugs targeting the sinusoidal endothelial cells in the liver.

## Figures and Tables

**Table 1:** Physicochemical properties of FPMs.

Polymeric Micelle	Hydrodynamic Diameter (nm)	PDI	Zeta Potential (mV)
FPM-CD32b	203.4 ± 3.6	0.44 ± 0.02	7.24 ± 0.21
FPM-CD36	258.2 ± 8.0	0.53 ± 0.12	6.16 ± 0.46
FPM-ITGB	179.4 ± 0.4	0.41 ± 0.01	−4.55 ± 0.59
FPM-ScrCD32b	288.0 ± 32.4	0.35 ± 0.02	6.88 ± 0.43

FPM: functionalized polymeric micelle; PDI: polydispersity index; Scr: scrambled. Values are indicated as mean ± SD.

**Table 2:** Hemodynamic measurements in 4-week BDL rats after 1 week of daily treatment.

Group	*n*	MAP (mmHg)	SMABF ((mL/min)·100 g)	SMAR ((mmHg·min)/ (mL·100 g))	PP (mmHg)	PBF ((mL/min)·100 g)	IHVR ((mmHg·min)/ (mL·100 g))	Heart Rate (BPM)
Untreated	5	90.75 ±3.86	4.09 ±0.82	19.09 ±2.20	19.73 ±0.41	3.17 ±0.71	7.46 ±1.75	327.2 ±19.35
Oral simvastatin	5	100.10 ±7.61	3.60 ±0.39	23.97 ±2.19	15.81 ±0.63 **^#^	2.56 ±0.32	6.51 ±0.79	332.5 ±11.67
PM-Sim	7	97.97 ±4.90	3.72 ±0.42	23.86 ±4.46	18.61 ±0.51	2.80 ±0.33	7.44 ±1.17	345.3 ±10.89
FPM-CD32b-Sim	6	91.50 ±4.56	4.28 ±0.78	20.31 ±3.42	17.81 ±0.74	2.62 ±0.46	7.84 ±1.30	321.0 ±8.30
FPM-CD36-Sim	4	89.95 ±4.14	4.43 ±0.56	17.26 ±2.86	18.20 ±0.68	3.43 ±0.07	5.31 ±0.27	332.4 ±14.32
FPM-CD32b-CD36-Sim	7	92.71 ±2.58	4.75 ±0.60	17.74 ±2.77	17.09 ±0.67	3.75 ±0.71	6.13 ±1.66	326.4 ±12.34

FPM: functionalized polymeric micelle; IHVR: intrahepatic vascular resistance; MAP: mean arterial pressure; PBF: portal blood flow; PM: polymeric micelle; PP: portal pressure; Sim: simvastatin; SMABF: superior mesenteric artery blood flow; SMAR: superior mesenteric artery resistance. Values were taken 90 min after the last dose of treatment and are expressed as mean ± SEM. *n* = number of rats. ** *p* ≤ 0.01 vs. untreated; # *p* ≤ 0.05 vs. PM-Sim.

**Table 3:** Hemodynamic measurements in week TAA-induced cirrhotic rats after a 2-weeks treatment (5 days/week).

Group	*n*	MAP (mmHg)	SMABF ((mL/min)·100 g)	SMAR ((mmHg·min)/ (mL·100 g))	PP (mmHg)	PBF ((mL/min)·100 g)	IHVR ((mmHg·min)/ (mL·100 g))	Heart Rate (BPM)
Untreated	7	106.20 ±5.18	4.60 ±0.57	22.37 ±2.86	11.96 ±0.49	3.84 ±0.22	3.18 ±0.24	341.0 ±9.42
Oral simvastatin	7	100.66 ±3.43	4.31 ±0.45	22.12 ±2.36	11.39 ±0.76	3.79 ±0.20	3.11 ±0.33	332.6 ±12.52
PM-Sim	6	96.93 ±3.11	4.84 ±0.88	20.40 ±2.84	10.22 ±0.38	4.70 ±0.63	2.34 ±0.27	335.6 ±6.55
FPM-CD32b-Sim	7	98.62 ±5.26	5.49 ±0.89	19.17 ±3.92	9.64 ±0.27 **^#^^	4.07 ±0.53	2.56 ±0.26	338.1 ±7.16
FPM-CD36-Sim	7	94.58 ±3.11	4.71 ±0.37	18.28 ±1.63	11.52 ±0.55	4.41 ±0.41	2.72 ±0.21	349.6 ±7.53
FPM-CD32b-CD36-Sim	6	92.42 ±3.96	4.19 ±0.41	20.80 ±2.54	10.15 ±0.52 *	4.13 ±0.19	2.47 ±0.11	321.8 ±13.25

FPM: functionalized polymeric micelle; IHVR: intrahepatic vascular resistance; MAP: mean arterial pressure; PBF: portal blood flow; PM: polymeric micelle; PP: portal pressure; Sim: simvastatin; SMABF: superior mesenteric artery blood flow; SMAR: superior mesenteric artery resistance. Values were taken 90 min after the last dose of treatment and are expressed as mean ± SEM. *n* = number of rats. * *p* ≤ 0.05, ** 0.01 vs. untreated; # *p* ≤ 0.05 vs. oral simvastatin; ^ *p* ≤ 0.05 vs. FPM-CD36-Sim.

## Data Availability

Data and analytic methods will be made available to other researchers at maria.martell@vhir.org.

## Supplementary Materials

The following supporting information can be downloaded at: https://www.mdpi.com/article/10.3390/pharmaceutics15102463/s1, Table S1. Biochemical parameters of 4-week BDL rats after 1-week daily treatment. Table S2. Biochemical parameters in 8-week TAA-induced cirrhotic rats after a 2-weeks treatment (5 days/week). Table S3. Reference average values of biochemical and hemodynamic parameters from healthy rats.
