# Supplementary material for: Optimization of Statin-Loaded Delivery Nanoparticles for Treating Chronic Liver Diseases by Targeting Liver Sinusoidal Endothelial Cells
Source: Pharmaceutics. 2023 Oct 14;15(10):2463. doi: 10.3390/pharmaceutics15102463 (PMC11340786; doi:10.3390/pharmaceutics15102463)
Supplement: Supplementary file 1 [file pharmaceutics-15-02463-s001.zip › pharmaceutics-2638303-supplementary.pdf]

SUPPLEMENTARY MATERIAL

Supplementary Table S1. Biochemical parameters of 4-week BDL rats after 1-week daily treatment

Group	n	Creatinine (mg/dl)	Bilirubin (mg/dl)	AST (IU/L)	ALT (IU/L)	ALP (IU/L)	CK (IU/L)	Cholesterol (mg/dl)	Triglycerides (mg/dl)	Albumin (g/dl)
**Untreated**	7	0.59<br>± 0.05	11.85<br>± 0.72	703<br>± 68.75	100<br>± 12.38	412<br>± 36.13	485<br>± 61.13	100<br>± 7.60	158<br>± 18.18	2.3<br>± 0.13
**Oral simvastatin**	11	0.54<br>± 0.07	10.26<br>± 0.53	1,426<br>± 316.31	187<br>± 47.09	439<br>± 44.80	974<br>± 264.31	158<br>± 22.54	195<br>± 40.42	2.4<br>± 0.11
**PM-Sim**	9	0.50<br>± 0.08	10.97<br>± 0.64	915<br>± 152.33	112<br>± 17.90	360<br>± 39.76	461<br>± 59.78	171<br>± 18.18	406<br>± 38.73**##	2.3<br>± 0.10
**FPM-CD32b-Sim**	8	0.59<br>± 0.07	11.73<br>± 0.48	1,135<br>± 267.24	148<br>± 25.02	482<br>± 35.95	628<br>± 115.93	201<br>± 19.65	451<br>± 56.45**##	2.3<br>± 0.10
**FPM-CD36-Sim**	9	0.60<br>± 0.04	11.19<br>± 0.98	1,144<br>± 355.95	106<br>± 29.61	405<br>± 23.10	567<br>± 64.83	196<br>± 25.14	466<br>± 51.76***###	2.1<br>± 0.07
**FPM-CD32b-CD36-Sim**	8	0.68<br>± 0.10	12.13<br>± 1.03	618<br>± 86.28	89<br>± 16.45	518<br>± 56.97	455<br>± 49.01	244<br>± 33.33**	548<br>± 61.22***###	2.2<br>± 0.08

ALP: alkaline phosphatase, ALT: alanine aminotransferase, AST: aspartate aminotransferase, CK: creatine kinase, FPM: functionalized polymeric micelle, PM: polymeric micelle, Sim: simvastatin. Values were taken during fasting and are expressed as mean ± SEM. n = number of rats. ** p≤0.01, *** 0.001 vs. untreated; ## p≤0.01, ### 0.001 vs. oral simvastatin

Supplementary Table S2. Biochemical parameters in 8-week TAA-induced cirrhotic rats after a 2-weeks treatment (5 days/week).

Group	n	Creatinine (mg/dl)	Bilirubin (mg/dl)	AST (IU/L)	ALT (IU/L)	ALP (IU/L)	CK (IU/L)	Cholesterol (mg/dl)	Triglycerides (mg/dl)	Albumin (g/dl)
**Untreated**	9	0.56<br>± 0.03	0.22<br>± 0.04	177<br>± 18.18	53<br>± 6.37	178<br>± 12.63	1,074<br>± 185.36	62<br>± 3.59+	33<br>± 1.63	3.0<br>± 0.10
**Oral simvastatin**	10	0.50<br>± 0.04	0.16<br>± 0.02	275<br>± 56.91	67<br>± 15.63	218<br>± 18.67	1,360<br>± 197.30	67<br>± 3.94	37<br>± 5.52	3.2<br>± 0.12
**PM-Sim**	10	0.50<br>± 0.06	0.27<br>± 0.05	166<br>± 10.65	45<br>± 5.84	188<br>± 32.07	882<br>± 185.70	76<br>± 3.78	123<br>± 46.65**##	3.1<br>± 0.13
**FPM-CD32b-Sim**	10	0.50<br>± 0.05	0.38<br>± 0.05*##+	225<br>± 34.30	53<br>± 4.57	193<br>± 25.36	1,515<br>± 258.87	62<br>± 3.53+	67<br>± 9.46***##+	3.0<br>± 0.12
**FPM-CD36-Sim**	10	0.54<br>±0.06	0.20<br>± 0.02	156<br>± 11.76	40<br>± 3.20	176<br>± 16.96	868<br>± 142.94	80<br>± 4.55*	43<br>± 4.88	3.1<br>± 0.10
**FPM-CD32b-CD36-Sim**	9	0.50<br>± 0.03	0.23<br>± 0.03	199<br>± 19.67	57<br>± 5.71	235<br>± 30.27	1,055<br>± 218.67	59<br>± 3.27^++	54<br>± 6.62**#	3.2<br>± 0.11

ALP: alkaline phosphatase, ALT: alanine aminotransferase, AST: aspartate aminotransferase, CK: creatine kinase, FPM: functionalized polymeric micelle, PM: polymeric micelle, Sim: simvastatin. Values were taken during fasting and are expressed as mean ± SEM. n = number of rats. * p≤0.05, ** 0.01, *** 0.001 vs. untreated; # p≤0.05, ## 0.01 vs. oral simvastatin; ^ p≤0.05 vs. PM-Sim; + p≤0.05, ++ 0.01 vs. FPM-CD36-Sim.

Supplementary Table S3. Reference average values of biochemical and hemodynamic parameters from healthy rats.

Biochemical parameter	Reference value (Healthy rats)
**Creatinine (mg/dl)**	0.40 ± 0.02
**Bilirubin (mg/dl)**	0.10 ± 0.00
**AST (IU/L)**	184 ± 23.83
**ALT (IU/L)**	44 ± 3.41
**ALP (IU/L)**	311 ± 17.79
**CK (IU/L)**	1,661 ± 506.71
**Cholesterol (mg/dl)**	79 ± 4.56
**Triglycerides (mg/dl)**	35 ± 9.30
**Albumin (g/d)**	2.9 ± 0.06

Hemodynamic parameter	Reference value (Healthy rats)
**MAP (mmHg)**	105.24 ± 6.55
**SMABF ([ml/min]·100 g)**	5.72 ± 0.59
**SMAR ([mmHg·min]/[ml·100 g])**	18.13 ± 2.70
**PP (mmHg)**	9.18 ± 0.60
**PBF ([ml/min]·100 g)**	5.03 ± 0.66
**IHVR ([mmHg·min]/[ml·100 g])**	2.01 ± 0.26
**Heart rate (BPM)**	312.7 ± 9.94

ALP: alkaline phosphatase, ALT: alanine aminotransferase, AST: aspartate aminotransferase, CK: creatine kinase. IHVR: intrahepatic vascular resistance, MAP: mean arterial pressure, PBF: portal blood flow, PP: portal

Protein analysis by Western blot: Figure 9.

Western blot images shown in figure 9 are the original untouched ones, with all samples used in the quantification and only slightly cropped in the margins. Sample groups are indicated on top of the blots to better define samples from different groups. The electrophoretic bands of Western blots were detected by digital chemiluminescence development using an Odyssey® Fc Imaging System (LI-COR Biosciences, Lincoln, USA). Protein quantification was done with Image Studio™ Lite software (LI-COR Biosciences, USA). The molecular marker (ladder) used in our western blots (Precision Plus Protein Standards, Dual Colors, Biorad Cat# 161-0374) is not chemiluminescent and is visualized as white bands in another wave length than the protein bands. The visualization system can also generate pdf documents where the blots, merged with ladders, are shown on the screen along with the exposure time, channel and file name. In every merged image, the first lane to the left, contains the molecular weight marker.
